# Altered pathways in methylome and transcriptome longitudinal analysis of normal weight and bariatric surgery women

**DOI:** 10.1038/s41598-020-60814-9

**Published:** 2020-04-15

**Authors:** C. F. Nicoletti, M. A. S. Pinhel, N. Y. Noronha, B. A. de Oliveira, W. Salgado Junior, A. Jácome, A. Diaz-Lagares, F. Casanueva, A. B. Crujeiras, C. B. Nonino

**Affiliations:** 1 Laboratory of Nutrigenomics Studies, Health Science Department, Ribeirão Preto Medical School, University of Sao Paulo, Ribeirão Preto, Brazil; 2 Laboratory of Studies in Biochemistry and Molecular Biology, Department of Molecular Biology, São José do Rio Preto Medical School, São José do Rio Preto, Brazil; 3 Department of Surgery and Anatomy, Ribeirão Preto Medical School, University of Sao Paulo, Sao Paulo, Brazil; 4 Department of Mathematics, MODES group, CITIC, Universidade da Coruña, Faculty of Science, A Coruña, Spain; 5 Translational Medical Oncology (Oncomet), Instituto de Investigación Sanitaria (IDIS), Complejo Hospitalario Universitario de Santiago (CHUS), CIBERONC, Santiago de Compostela, Spain; 6 Roche-CHUS Joint Unit, University Clinical Hospital of Santiago (CHUS), Santiago de Compostela, Spain; 7 Epigenomics in Endocrinology and Nutrition, Instituto de Investigación Sanitaria (IDIS), Complejo Hospitalario Universitario de Santiago (CHUS) and Santiago de Compostela University (USC), Santiago de Compostela, Spain; 8 CIBER Fisiopatología de la Obesidad y la Nutrición (CIBERobn), Madrid, Spain

**Keywords:** Molecular medicine, Predictive markers

## Abstract

DNA methylation could provide a link between environmental, genetic factors and weight control and can modify gene expression pattern. This study aimed to identify genes, which are differentially expressed and methylated depending on adiposity state by evaluating normal weight women and obese women before and after bariatric surgery (BS). We enrolled 24 normal weight (BMI: 22.5 ± 1.6 kg/m^2^) and 24 obese women (BMI: 43.3 ± 5.7 kg/m^2^) submitted to BS. Genome-wide methylation analysis was conducted using Infinium Human Methylation 450 BeadChip (threshold for significant CpG sites based on delta methylation level with a minimum value of 5%, a false discovery rate correction (FDR) of q < 0.05 was applied). Expression levels were measured using HumanHT-12v4 Expression BeadChip (cutoff of p ≤ 0.05 and fold change ≥2.0 was used to detect differentially expressed probes). The integrative analysis of both array data identified four genes (i.e. *TPP2*, *PSMG6*, *ARL6IP1* and *FAM49B*) with higher methylation and lower expression level in pre-surgery women compared to normal weight women: and two genes (i.e. *ZFP36L1* and *USP32*) that were differentially methylated after BS. These methylation changes were in promoter region and gene body. All genes are related to MAPK cascade, NIK/NF-kappaB signaling, cellular response to insulin stimulus, proteolysis and others. Integrating analysis of DNA methylation and gene expression evidenced that there is a set of genes relevant to obesity that changed after BS. A gene ontology analysis showed that these genes were enriched in biological functions related to adipogenesis, orexigenic, oxidative stress and insulin metabolism pathways. Also, our results suggest that although methylation plays a role in gene silencing, the majority of effects were not correlated.

## Introduction

DNA methylation in CpG dinucleotides is a dynamic process and best characterized epigenetic modifications^1^. Methylation patterns are established in early life and can be remodeled in adult cells, by modulating DNA interactions with proteins and transcription factors^2,3^, being able to alter gene expression profile^4^.

Weight gain or loss during infancy and adulthood, by changing the energy storage and adipose tissue homeostasis, may alter molecular mechanisms and biological processes^5^. In line of this, epigenetic changes may predispose a disease risk or can occur once a disease has developed^6^. Thus, DNA methylation provides a link between environmental, genetic factors and weight control.

Obesity has reached epidemic proportions and, in 2016, affected about 1.9 billion adults worldwide^7^, being associated to a public health burden. In the context of obesity treatment, bariatric surgery (BS) has been the best choice for cases of severe obesity and has been shown to be the most effective way to promote significant and sustained weight loss^8^. Among the surgical techniques, Roux-en-Y gastric bypass (RYGB) is the most performed in Brazil and worldwide, and is considered a gold standard because its high efficacy and low morbid-mortality^9^. However, inter-individual variability of the response to bariatric surgery, mainly related to weight loss, is evident^10^, and the unpredictability of weight-loss success represents a significant barrier in the surgical management of obesity^11^.

Recent epigenome-wide association studies (EWAS) evidenced multiple DNA methylation loci associated with body mass index (BMI)^12,13^. In addition, the role of epigenetic signature in metabolic results after obesity treatment has been described previously^14,15^. Knowing that nutrition is one of the greatest environmental stimuli that can change gene methylation/expression signature^16^, some studies have demonstrated that BS changes DNA methylation levels^17^. Other publications have evidenced that weight loss by surgery is also able to modify gene expression levels, mainly of genes related to metabolic pathways^18,19^.

The relevance of differential DNA methylation and gene expression consequent of BS was previously evidenced by our research group^20,21^; however, to gain a broader perspective about the interface between CpG methylation and functional effects in transcription, we performed an integrated analysis with methylation and expression data. Thus, the objective of the current study was to identify genes, which are differentially expressed and methylated depending on adiposity state by evaluating normal weight women and obese women before and after BS.

## Results

### Sample characteristics

No significant difference was found between the ages of each group (obese patients: 36.9 ± 10.2 years and normal weight women: 36.9 ± 11.8 years; p > 0.05). Normal weight women showed a mean weight of 60.7 ± 6.3 kg and mean fat mass of 29.6 ± 4.1%. After 6 months of BS, we evidenced a significant weight (113.3 ± 16.3 to 86.3 ± 12.5 kg, *p* < 0.001), BMI (43.3 ± 5.7 to 33.1 ± 4.8 kg/m^2^, *p* < 0.001) and fat mass (45.7 ± 5.7 to 36.1 ± 5.9%, *p* < 0.001) reduction.

### Comparison of gene expression and methylation patterns and integrated analysis of the data

From the genome-wide DNA methylation study, 1,074 CpG sites (located in 769 genes) were differentially methylated between obese women before BS and normal weight women, and most of them showed high methylation level in obese patients. Also, 666 CpGs (located in 495 genes) had methylation levels changed after RYGB. For the microarray gene expression analysis, a total of 600 genes were differently expressed (DEGs) between obese women before BS and normal weight patients. Indeed, 1,366 genes were differentially expressed between surgery times. Among these, 88 genes were up- and 512 genes were down-regulated.

**Figure 1:**
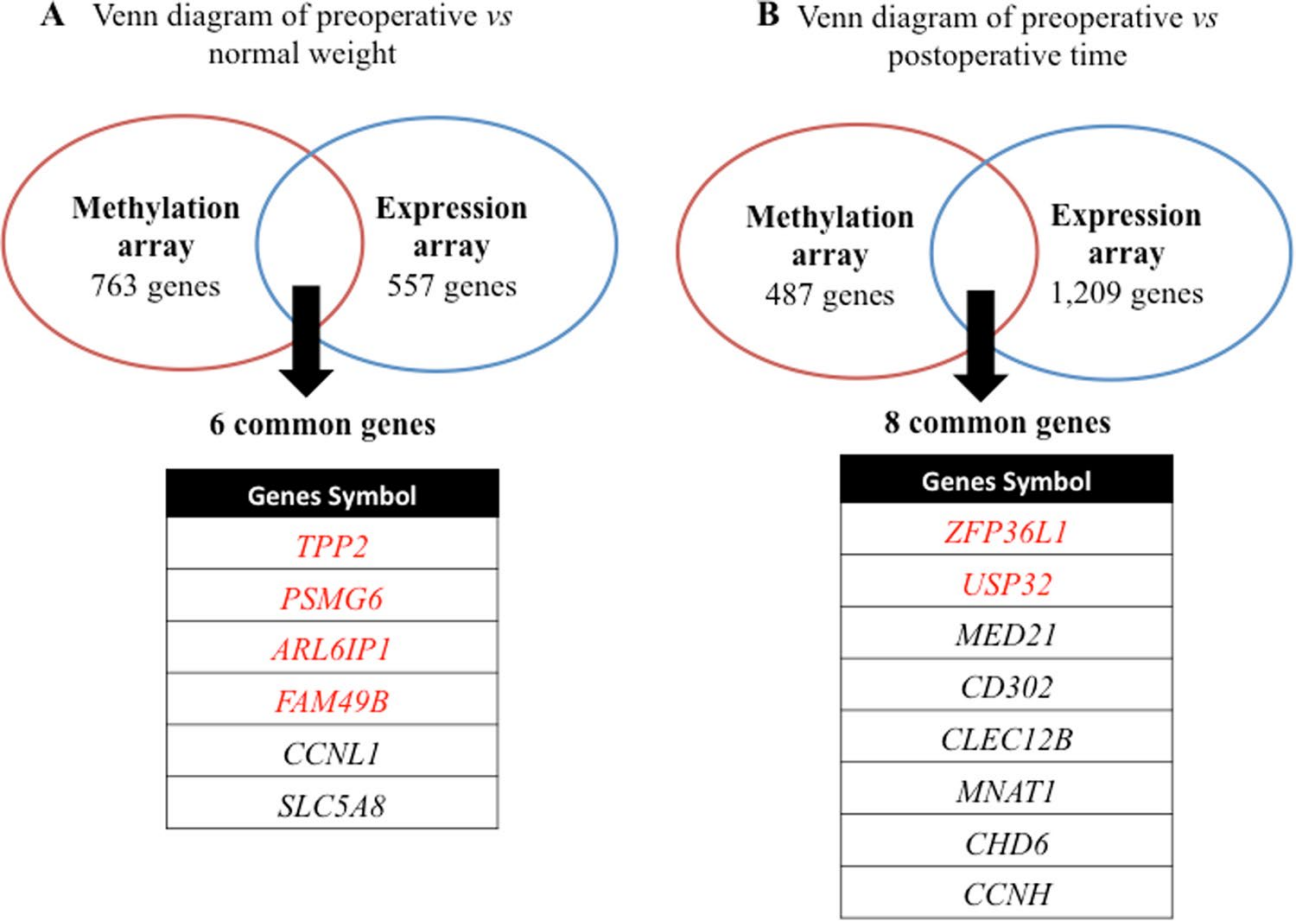
Clustering analysis of the methylation and expression arrays. (**A**) Venn diagram of genes detected in the pre-surgery versus the normal weight patients; (**B**) Venn diagram of genes detected in pre-surgery period versus the post-surgery period.

Figure 1 illustrates the Venn diagram showing the overlap of differently expressed genes (fold change >2, FDR *p* < 0.05) and differently methylated CpG sites (Δβ ± 5%, *p* < 0.001). When preoperative patients and normal weight women were compared, a total of six genes were, concomitantly, differentially methylated and expressed (Fig. 1A). Two of them (*CCNL1* and *SLC5A8*) present their methylation and expression levels in the same direction: both lower in preoperative patients compared to normal weight women. However, four genes (*TPP2*, *PSMG6*, *ARL6IP1* and *FAM49B*) presented methylation and expression levels in opposite direction: higher methylation and lower expression level in pre-surgery women compared to normal weight women. Also, these CpG sites were located at TSS200, TSS1500 and 5′UTR regions (gene promoter region).

Considering pre and postoperative times comparison, Venn diagram analysis identified a total of eight genes that were common in methylation and expression arrays (Fig. 1B). Of these, two genes (*ZFP36L1* and *USP32*) were differentially methylated and expressed after RYGB. Methylation and expression levels of *ZFP36L1* and *USP32* genes were changed in opposite direction. For *ZFP36L1*, the methylation level decreased after surgery while its expression increased. In contrast, for *USP32*, methylation level increased and expression decreased after the surgery. Considering CpGs location, we observed that they were located in gene body and TSS1500 region.

The expression analysis between postoperative and normal weight individuals showed no differential gene expression, thus concomitant analysis between methylation and expression profile couldn’t be performed.

Table 1 summarizes the results of integrated analysis between DNA methylation and gene expression arrays, that is, gene concomitantly differently methylated and expressed between groups (obese and normal weight women) and periods (before and after BS).

**Table 1:** Genes overrepresented identified from differential methylation and expression arrays.

Obese patients before bariatric surgery *versus* normal weight women						
Gene	Fold change (OB-NW)	CpG name	CpG region	Δβ (OB-NW)	Integrated results	Gene Ontology (by GOA)
*TPP2*	−0.42	cg02184819	PR	0.057	+methylated on obese −expressed on obese	proteolysis protein polyubiquitination
*PSMC6*	−0.23	cg00686598	PR	0.094	+methylated on obese −expressed on obese	MAPK cascade NIK/NF-kappaB signaling
*ARL6IP1*	−0.23	cg08040170	PR	0.076	+methylated on obese −expressed on obese	apoptotic process cotranslational protein targeting to membrane
*FAM49B*	−0.44	cg02711724	Body	0.107	+methylated on obese −expressed on obese	platelet degranulation positive regulation of T cell activation
*CCNL1*	−0.30	cg02399570	Body	−0.093	−methylated on obese −expressed on obese	positive regulation of cyclin-dependent protein serine/threonine kinase activity
*SLC5A8*	−0.49	cg06162589	Body	−0.055	−methylated on obese −expressed on obese	NAD biosynthesis via nicotinamide riboside salvage pathway short-chain fatty acid import
**Before** ***versus*** **after bariatric surgery**						
**Gene Symbol**	**Fold change (OB-NW)**	**CpG name**	**CpG region**	**Δβ (OB-NW)**	**Integrated results**	**Gene function (by GOA)**
*ZFP36L1*	2.18	cg08169020	Body	0.077	−methylated after RYGB +expressed after RYGB	MAPK cascade cellular response to insulin stimulus
*USP32*	0.40	cg00691729	Body	0.076	+methylated after RYGB −expressed after RYGB	protein deubiquitination ubiquitin-dependent protein catabolic process
*MED21*	7.58	cg01982833	PR	0.074	+methylated after RYGB +expressed after RYGB	positive regulation of transcription by RNA polymerase II stem cell population maintenance
*CD302*	3.25	cg03432176	PR	0.069	+methylated after RYGB +expressed after RYGB	phagocytosis signal transduction
*CLEC12B*	4.19	cg05849676	Body	0.069	+methylated after RYGB +expressed after RYGB	negative regulation of signaling receptor activity natural killer cell inhibitory signaling pathway
*MNAT1*	2.28	cg07212327	Body	0.055	+methylated after RYGB +expressed after RYGB	DNA repair adult heart development
*CHD6*	2.14	cg00514723	PR	0.051	+methylated after RYGB +expressed after RYGB	positive regulation of transcription from RNA polymerase II promoter in response to oxidative stress viral process
*CCNH*	2.18	cg02021919	Body	0.087	+methylated after RYGB +expressed after RYGB	protein phosphorylation positive regulation of cyclin-dependent protein serine/threonine kinase activity

OB: obese women; NW: normal weight women; Methylation data showed in Δβ; Expression data showed in Fold change; GOA: Gene Ontology Annotation; *TPP2*: tripeptidyl peptidase 2; *PSMC6*: proteasome 26S subunit, ATPase 6; *ARL6IP1*: ADP ribosylation factor like GTPase 6 interacting protein 1; *FAM49B*: family with sequence similarity 49 member B; *CCNL1*: cyclin L1; *SLC5A8*: solute carrier family 5 member 8; *ZFP36L1:* ZFP36 ring finger protein like 1; *USP32:* ubiquitin specific peptidase 32; *MED21*: mediator complex subunit 21; *CD302:* CD302 molecule; *CLEC12B*: C-type lectin domain family 12 member B; *MNAT1*: CDK activating kinase assembly fator; *CHD6*: chromodomain helicase DNA binding protein 6; *CCNH*: cyclin H; PR: promoter region (TSS200, TSS1500, 5′UTR). *p* < 0.001 for all analysis.

To additional understanding about the biological relevance of the identified genes, GO analyses were performed. These analyses showed that these genes are related to MAPK cascade, NIK/NF-kappaB signaling, cellular response to insulin stimulus, proteolysis and others.

## Discussion

We identified in the present study genes that are concomitant differentially methylated and expressed in leukocytes after BS. Relevantly, these genes were involved in molecular pathways related to obesity´s physiopathology. Also, comparing obese subjects (before BS) and normal weight women, we identified four genes that showed lower expression and higher methylation levels; which were related to adipogenesis, anti-satiety effect, oxidative stress, and insulin metabolism. Moreover, we evidenced that two genes related to proteolysis and adipogenesis, were epigenetically regulated by RYGB procedure.

Despite the fact that the impact of DNA methylation on gene expression seems to depend of cytosine methylated site and location^22,23^, results obtained here showed that promoter methylation (TSS200 and TSS1500) is associated with decreased expression; however, for specific CpG sites, intragenic methylation (gene body) also correlates with decreased gene expression. Grundberg *et al*.^24^, found that a large number of methylation and expression associations were positive, thus the increase in methylation levels was linked to an increase of corresponding gene expression. More interestingly, evidence suggested there an adjustment mechanism of body gene methylation levels for transcription regulation. In line of this, when promoter methylation is constant, increasing body methylation is associated with more repressed expression^25^. Thus, considering that some authors affirmed that the function of DNA methylation in intergenic and gene-body regions is less defined^24^, our results showed that regardless of the location of methylated cytosine (promoter region or body), DNA methylation promotes a reduction in gene expression.

In addition, it is important to highlight that the expression of two (*CCNL1* and *SLC5A8)* and six genes (*MED21*, *CD302*, *CLEC12B*, *MNAT1, CHD6* and *CCNH*) were not regulated by DNA methylation in obese *versus* normal weight and preoperative *versus* postoperative comparisons, respectively. Study with cancer cells showed that the presence of methylation does not always imply in gene silencing^26^. Thus, our results showed that despite methylation plays a role in gene silencing, the majority of effects were not correlated.

Another point to be discussed is that the present study is the first to identify an association between *TPP2*, *PSMC6*, *ARL6IP1* and *FAM49B* genes with obesity. The *TPP2* gene codifies a peptidase that has been related to an anti-satiety effect by degrading the cholecystokinin 8 hormone (CCK8) and adipogenesis stimulus, pointing to an important role of *TPP2* in obesity^27,28^. Thus, *TPP2* seems to have dual role in metabolic homoeostasis, affecting both feeding behavior and adipose tissue biology *per se*^28^. Moreover, TPP2 is also related to different isoforms of protein convertase gene family (PSCK) that are associated with obesity related traits (e.g PCSK1 and PCSK2)^29^.

On the other hand, *ARL6IP1* and *PSMC6* have been associated with insulin synthesis and pathology of diabetes, respectively^30,31^. Also, *ARL6IP1* protein is associated phosphatidylethanolamine-binding family of proteins (PEBP1), which has important role in MAPK, NF-kappa B, and glycogen synthase kinase-3 (GSK-3) signaling pathways. *PSMC6* gene encodes one of the ATPase subunits and its expression was inversely correlated with BMI^11^.

Lastly, *FAM49B* has been appointed as novel regulator of mitochondrial function and may thus be associated with oxidative stress and inflammation, however, up to now, no functional data about this protein has been published^32^. Furthermore, *USP32* is a highly conserved gene and uncharacterized gene that its stable silencing caused a significant decrease in the proliferation and migration rate of cells^9^. For this reason, *USP32* has been associated with growth rate of cancer cells^9,33^.

We also identified that bariatric surgery modify *ZFP36L1* and *USP32* methylation and expression levels in leukocytes. In culture 3T3-L1 preadipocytes, *ZFP36L1* was associated with adipogenesis^34^ by regulating adipogenesis rate, playing an important role in obesity development^35^. According to Tseng^36^, *ZFP36L1* overexpression might repress adipogenesis at least by down-regulating *PPARG2* expression. These findings are a proof that blood leukocytes are able to reflect the regulation of same adipose tissue biology-related genes as it was also demonstrated in previous reports^13,37–39^. In the current research, we were unable to detect differences in methylation levels of previously identified obesity related CpG sites, probably because differences in the study design, sample size or the threshold used for selection of candidates. However, the current study adds new information to this issue. In fact, the identified genes were involved in adipose tissue-related pathways that were also observed in previous reports on obesity-related methylation profile^13,38,39^.

The use of DNA and RNA extracted from leukocytes is a limitation of this study because both, expression and methylation, are tissue specific molecular mechanisms. However, leukocytes samples are less invasive, more convenient and acceptable than biopsies of target tissues (i.e adipose tissue) to perform longitudinal assessments and more suitable in clinical practice. To search for obesity-associated epigenetic biomarkers for the diagnosis and management of the disease is a huge challenge as adipose tissue is inaccessible without surgery. As mentioned above, in the obesity field, relevant studies have been recently published providing evidence that blood cells can be used to identify robust and biologically relevant epigenetic variation related to BMI. Moreover, it was demonstrated that epigenetic biomarkers in blood could mirror age-related epigenetic signatures in biologically relevant target tissues such as adipose tissue. Therefore, blood leukocytes are suitable, minimally invasive biological sources to evaluate obesity signatures. On the other hand, even though the identified changes in methylation and expression pattern induced by bariatric surgery in the present study could be transient, long-term studies would indicate the degree of plasticity of the epigenomic changes associated with bariatric surgery and would establish if over a longer time, the methylation profiles returned to the obese-pre surgery levels.

In conclusion, integrating analysis of DNA methylation and gene expression evidenced that there is a set of genes relevant to obesity that changed after BS. A gene ontology analysis showed that these genes were enriched in biological functions related to adipogenesis, orexigenic, oxidative stress and insulin metabolism pathways. Also, our results suggest that although methylation plays a role in gene silencing, the majority of effects were not correlated.

## Methods

### Study participants

The present study included 24 normal weight women (BMI: 22.5 ± 1.6 kg/m^2^, 36.9 ± 11.8 years) and 24 severe obese women (BMI: 43.3 ± 5.7 kg/m^2^, 36.9 ± 10.2 years) submitted to RYGB which methylation and gene expression profile had been analyzed previously^20,21^. All samples from obese women were collected at preoperative time and after 6 months of bariatric surgery. Normal weight women were evaluated once.

All obese participants underwent open performed RYGB surgery that consisted in creating a small gastric portion (30 to 50 mL) and an anastomosis of the gastric stump to the jejunum (both remaining loops measured about 100 cm). The surgical procedure was standardized at our hospital following the most usual pattern performed worldwide and well described^40^. There were not postoperative complications in the patients included in this study.

The study was conducted with the approval of the Hospital Ethics Committee and in agreement with the Declaration of Helsinki. Informed consent was obtained from all individual participants included in the study. All methods were carried out in accordance with relevant guidelines and regulations.

### DNA methylation array analysis and data processing

As aforementioned^21^, genomic DNA was extracted from peripheral mononuclear blood cells (PMBC) using GE Health Care kit (Illustra blood genomic Prep Mini Spin kit) and this extracted DNA was stored at −80 °C until the next steps. DNA fragmentation or RNA contamination was analyzed by 1% agarose gel electrophoresis. Also, DNA was bisulfite converted using EZ DNA methylation kit Methylation-Gold (Zymo Research, CA, USA) according to the manufacturer’s instructions and then immediately hybridized in BeadChip. Genome-wide methylation analysis was conducted using the Infinium Human Methylation 450 BeadChip (Illumina, San Diego, CA, USA). Beadchips were scanned with the Illumina iScanSQ system and DNA quality checks, bisulfite modification, hybridization, data normalization, and statistical filter were performed as described before^21^.

Analysis of methylation data were conducted using the Genome Studio software version 2011.1 (Illumina Inc.) and methylation levels were expressed in beta values (β). β values that were calculated as the intensity of the methylated channel divided by the total intensity (β = Max (SignalB, 0)/(Max (SignalA, 0) + Max (SignalB, 0) + 100), and ranged from 0 (unmethylated) to 1 (fully methylated). β values with detection p-values > 0.01 were removed from analysis because were considered to fall below the minimum intensity and threshold. Also, probes that were localized to the sex chromosomes and those CpGs that contained single nucleotide polymorphisms were filtered out.

Differences in methylation levels between preoperative and postoperative groups (Δβ) was calculated for a given CpG site by subtracting the mean beta value from the pool of pre-surgery samples (pre-surgery period) as compared to the pool of samples collected after six months of RYGB and were tested by t test. Values of *p* were adjusted for multiple comparisons by using the false discovery rate (FDR below 5% was considered statistically significant). Also, a threshold for the significant CpG sites based on Δβ with a minimum value of 5% (value greater than 0.05 or less than −0.05) and FDR < 0.05 was applied. We used R software (version 3.2.0) to perform these analyzes.

### Gene expression array analysis and data processing

Total RNA was extracted and purified from whole blood using phenol-chloroform extraction method modified by Chomczynski & Sacchi^41^. Extracted RNA was stored at −80 °C until the next steps. After, RNA integrity number (RIN) was analyzed using Bioanalyzer (Agilent Technologies, Cedar Creek, TX, USA). Expression levels were measured using the HumanHT-12 v4 Expression BeadChip (Illumina Inc.) Expression data were visualized and analyzed using Genome Studio Software (Illumina®). Beadchips were also scanned with the Illumina HiScanSQ system and Genome Studio Software (Illumina^®^) was also used. For quality control and normalization, the samples with less than 6000 significantly detected probes (detection p-value < 0.01) were excluded and differentially expressed transcripts were identified with 95% confidence of no more than 1% false positive using an Mann-Whitney test. A cutoff of p ≤ 0.05 was used to detect differentially expressed probes. In addition, a cutoff of fold change ≥2.0 (symmetrical fold change ≥2.0 or ≤ −2.0) was used. p values obtained from permutations and fold change cutoff values were then used to minimize the chances of false positives. Gene expression microarray results were validated using real-time polymerase chain reaction (RT-PCR; Applied Biosystems Gene Expression Assays; Applied Biosystems, Foster City, CA, USA)^20^.

For both methylation and expression analysis, samples from obese and normal weight women were randomly scattered on each Beadchip and not one batch was one and the other was opposite.

### Overlapping of pooled gene expression and DNA methylation analysis

**Figure 2:**
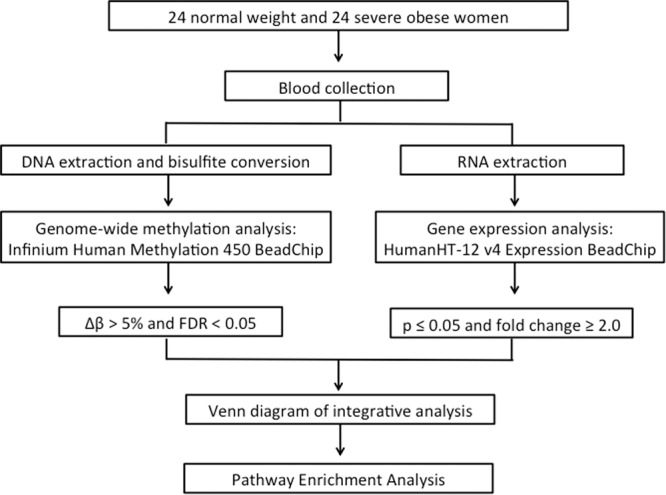
Study’s workflow of integrative analysis of methylation and expression arrays.

Before comparing gene expression and methylation analysis, a correction by cellular type was made in DNA methylation data (Supplementary Table 1). By crossing and comparing the differentially regulated gene (DEG) and differentially methylated genes (DMG), the list generated from the gene expression analysis^21^ were overlapped with the lists from the methylation analysis^20^ using Venn diagram (http://bioinfogp.cnb.csic.es/tools/venny/). Figure 2 shows a schematic diagram of integrative analysis of methylation and expression arrays.

### Pathway enrichment analysis

Overrepresented pathways were then obtained from this new list of common genes. Genes were classified according to gene function. For this, the WebGestalt program (Gene SeT AnaLysis based on the WEB, http://www.webgestalt.org) and KEGG signaling pathway analysis were used. The identification numbers (IDs) were loaded and analyzed against the human reference genome by means of a Bonferroni multiple test adaptation threshold of *p* < 0.05.

### Ethics approval and consent to participate

This study has been approved by the Ethical Committee of Clinical Hospital of Ribeirao Preto School of Medicine, University of São Paulo. All patients gave their written consent for participation in the study.

## Supplementary information

Supplementary Table 1.

## Data Availability

Illumina HumanHT-12 v4 Expression BeadChip data has been submitted to Gene Expression Omnibus (GEO) with accession number GS E83223 (https://www.ncbi.nlm.nih.gov/geo/query/acc.cgi?acc=GSE83223).
