# Supplementary material for: Altered pathways in methylome and transcriptome longitudinal analysis of normal weight and bariatric surgery women
Source: Sci Rep. 2020 Apr 15;10:6515. doi: 10.1038/s41598-020-60814-9 (PMC7160100; doi:10.1038/s41598-020-60814-9)
Supplement: Supplementary file 1 — Supplementary Table 1. [file 41598_2020_60814_MOESM1_ESM.docx]

**TITLE PAGE**

**Title:** Altered pathways in methylome and transcriptome longitudinal analysis of normal weight and bariatric surgery women

**Authors:** Nicoletti CF^1^; Pinhel MAS^1,2^; Noronha NY^1^; de Oliveira BA^1^; Jácome A^3^; Diaz-Lagares A^4^, Casanueva F^5^, Crujeiras AB^5^; Nonino CB^1^

**Filiation:**

^1^Laboratory of Nutrigenomics Studies, Health Science Department, Ribeirão Preto Medical School, University of Sao Paulo, Brazil.

^2^Laboratory of Studies in Biochemistry and Molecular Biology, Department of Molecular Biology, São José do Rio Preto Medical School, Brazil.

^3^Department of Mathematics, MODES group, CITIC, Universidade da Coruña, Faculty of Science, A Coruña, Spain.

^4^Translational Medical Oncology (Oncomet), Instituto de Investigación Sanitaria (IDIS), Complejo Hospitalario Universitario de Santiago (CHUS), CIBERONC, Santiago de Compostela, Spain; Roche-CHUS Joint Unit, University Clinical Hospital of Santiago (CHUS), Santiago de Compostela, Spain.

^5^Epigenomics in Endocrinology and Nutrition, Instituto de Investigación Sanitaria (IDIS), Complejo Hospitalario Universitario de Santiago (CHUS) and Santiago de Compostela University (USC), Santiago de Compostela, Spain; CIBER Fisiopatología de la Obesidad y la Nutrición (CIBERobn), Madrid, Spain.

**Corresponding author:**

Correspondence and requests for materials should be addressed to Carla Barbosa Nonino (email: carla@fmrp.usp.br) and Ana Belén Crujeiras (email: [anabelencrujeiras@hotmail.com](mailto:anabelencrujeiras@hotmail.com)).

| Supplementary Table 1. Cellular type among groups | | | |  |
| --- | --- | --- | --- | --- |
| Cellular type | Normal weight  women | Obese patients before bariatric surgery | Obese patients after bariatric surgery | |
| CDT8 | 58196285.9±40525623.1 | 59833971.3±23587022.6 | 81438220.3±39848750.3 | |
| CDT4 | 14006187.1±5263259 | 16899279.8±5328502.6 | 22224812.2±8649257.5 | |
| Natural killer | 26586.9±30348.4 | 45357.1±38842.1 | 72960.7±48664.4 | |
| B cells | 337374732.3±216925693.9 | 528167476.9±253243036.1 | 601939458±222850023 | |
| Monocitos | 76984272.1±26431235.7 | 78704338.6±26366420.2 | 51202572.8±25324147.4 | |
| Granulocitos | 6509307.1±955996.7 | 5883536.6±933495 | 5074985.8±1356656.9 | |
